# The Drunkard

**Published:** 1869-10

**Authors:** 


### THE DRUNKARD.

Very few persons would be willing to marry a habitual drunkard; but it is not generally known what a great risk is run in marrying the son or daughter of a habitual drunkard, although they themselves are strictly temperate from high moral principle; indeed, it is not an uncommon thing to find the children of beastly drunkards the very models of temperance, from having had before their own eyes, for years in succession, the terrible evils of habitual inebriation.

It is one of the indisputable facts in physiology, and the observations of intelligent men confirm the truth, that certain diseases and taints of body, and traits of mind are transmitted from father to son. So well and firmly is this impression fixed on the minds of men, that when a man becomes insane, one of the very first efforts is to endeavor to ascertain if it is not "in the family," and it is comparatively seldom that such is not proved to be the case.

Another important fact is, that hereditary traits and taints sometimes overleap a generation; arising most probably from the fact that one parent has extraordinary good health, sufficiently vigorous to stave off the malady for a time; but the seed of the malady is in the immediate descendant for all that, and to the extent, that if the grandchild marries one who has a similar taint, the offspring develops the characteristic of the grandparent.

Drunkenness is a transmissible malady, because anatomical investigations demonstrate that the brain of a drunkard, after a comparatively few indulgences, becomes organically impaired, and when that is the case, it is just as impossible to repair the injury as to have a new finger grow in the place of one which has been removed. Surely no stronger appeal can be made to a man's intelligence, to his honor, and to his humanity, to practice temperance in the use of all intoxicating drinks.

As a proof of the argument made, it may be sufficient to say in general terms, that observation shows, that, in any number of drunkards, about one-third become so through social influences, the remaining two-thirds from hereditary influences. More than half of the first class are reclaimed, but to recover men from intemperate habits, who have become so from hereditary influences, is almost impossible, even although they may have had a Christian education and the early instillment of strictly temperance principles. Let the reader who can, thank God that he has not had the curse of an intemperate parent, and let him pray daily, with consistent action, that he may never be permitted to fall into so great a crime as that of being an intemperate parent himself. Nor ought a man who has been a drunkard, to allow himself to marry and become the father of children, for they are very certain either to inherit his vice or to have implanted in their constitutions the seeds of insidious diseases. To be safe from these calamities and crimes there is only one safe plan, never taste a drop of the accursed thing.

